# Treatment outcomes of induction chemotherapy combined with intensity-modulated radiotherapy and adjuvant chemotherapy for locoregionally advanced nasopharyngeal carcinoma in Southeast China

**DOI:** 10.1097/MD.0000000000027023

**Published:** 2021-08-20

**Authors:** Peirong Wang, Feng Dong, Chuanshu Cai, Chunlin Ke

**Affiliations:** Department of Radiation Oncology, The First Affiliated Hospital of Fujian Medical University, No. 20, Chazhong Road, Taijiang District, Fuzhou, Fujian, China.

**Keywords:** adjuvant chemotherapy, induction chemotherapy, intensity-modulated radiotherapy, locoregionally advanced nasopharyngeal carcinoma, survival

## Abstract

Induction chemotherapy (IC) and adjuvant chemotherapy (AC) are used to enhance tumor locoregional control and support early treatment for distant metastases. However, optimum combinatorial treatment of these chemoradiotherapy regimens with radiotherapy in curing locoregionally advanced nasopharyngeal carcinoma (NPC) remains unclear. Here, we evaluate the efficacy and therapeutic outcome of a combinatorial treatment strategy involving IC, intensity-modulated radiotherapy (IMRT), and AC, by retrospectively analyzing 243 NPC patients who were treated by IC followed by IMRT and AC. The rates of 3-/5-year local-regional control rate, distant failure-free rate (DFFR), progression-free survival (PFS), and overall survival (OS) were 93.3%/90.3%, 84.2%/79.4%, 79.6%/74.4%, and 84.0%/72.6%, respectively. The 3-/5-year OS rates of patients in stage III or IVA were 91.5%/75.1% and 86.5%/56.5%, respectively. Combination cisplatin with paclitaxel showed no significance in OS as compared to cisplatin plus 5-fluorouracil (*P*-value = .17). Total four-cycle IC and AC was significantly beneficious versus three-cycle in DFFR (*P*-value = .04), as well as total 6 chemotherapy cycles compared to 4 in DFFR and PFS (*P*-value = .03 and *P*-value = .01, respectively). All survival indicators were adversely affected by T-category, while N-category could only predict DFFR and PFS. Radiation dosage represented as a second prognostic factor for local control. We propose that IC combined with IMRT and AC for locoregionally advanced NPC shows effective treatment outcomes.

## 1 Introduction

Nasopharyngeal carcinoma (NPC) is a regionally distributed disease, which shows the highest incidence in Southern China.^[1]^ Radiotherapy is the cornerstone treatment for NPC. As NPC is a kind of chemosensitive tumor, radiotherapy is usually combined with chemotherapy for locoregionally advanced NPC. Induction chemotherapy (IC) and post-radiotherapy adjuvant chemotherapy (AC) are used to enhance locoregional control and can support early treatment for occult micrometastases or distant metastases. As far as our knowledge; however, the optimum treatment sequence and combination of these chemotherapy regimens remains unclear. Previous studies provide 7 different combinatorial treatments, among which concurrent chemoradiotherapy (CCRT) is a standard choice for locoregionally advanced NPC.^[2]^ However, a growing body of studies show that CCRT provides no significant benefit compared to the treatment of intensity-modulated radiotherapy (IMRT) in curing locoregionally advanced NPC; but is responsible for high rates of 3- or 4-grade acute toxicities and late toxicities.^[3–6]^ Mucositis and weight loss are 2 most common characters in the patient during treatment by CCRT, which are associated with poorer tolerance and unfavorable efficacy of treatment.^[7]^ Thus, the additive benefit of IMRT combined with chemotherapy regimens is largely unknown. Further investigation on the sequence of using chemotherapies for NPC patients is needed in the IMRT-treated patients.

This study aims to evaluate the effectiveness of the combination of IC with IMRT and AC in treating locoregionally advanced NPC. We retrospectively analyzed 243 diagnosed patients with NPC from the medical center of Southeast China, The First Affiliated Hospital of Fujian Medical University. These clinical data represent a helpful treatment benchmark for concurrent chemotherapy with IMRT in treating NPC.

## 2 Materials and methods

This study was evaluated and approved by the ethics committee of the First Affiliated Hospital of Fujian Medical University and the informed consent was waived because this is a retrospective study and used the data anonymously. This study includes a total of 243 pathologically diagnosed NPC patients without distant metastases that were treated by definitive IMRT in the First Affiliated Hospital of Fujian Medical University. All patients underwent fiberoptic nasopharyngoscopy, physical examination, biochemical and hematological blood tests, magnetic resonance imaging of nasopharynx and neck, computed tomography scan of thorax and abdomen, and emission computed tomography. The 8th edition of the American Joint Committee on Cancer and International Union Against Cancer (AJCC/UICC) staging system was restaged for all patients.

IMRT was applied for all patients. Patients were immobilized with a thermoplastic head, neck, and shoulder mask in the supine position. Intravenous contrast-enhanced CT or not were used for planning, 3 mm slices from the calvarium through the tracheal bifurcation. The target volumes were defined in accordance with the International commission on Radiation Units and Measurements reports 50 and 62. The primary nasopharyngeal gross tumor volumes (GTVnx) and the positive lymph nodes (GTVnd) were determined by imaging, clinical, and endoscopic findings. The first clinical target volume 1 was defined as GTVnx plus a margin of 5 to 10 mm and encompassed high-risk region. The second clinical target volume 2 was defined as clinical target volume 1 plus a margin of 5 to 10 mm and encompassed low-risk region. Clinical target volume 2 is close to critical organs, such as the brainstem and spinal cord; the margin was reduced to 3 to 5 mm. Planning target volume would encompass clinical target volume with a 3-mm margin in all directions. The critical organs include the spinal cord, brain stem, optic nerves, optic chiasm, temporal lobes, lens, eyeballs, parotid glands, temporomandibular joints, thyroid gland, and pituitary. The most common fractionation schedule was 2.12 Gy (range, 2.0–2.2 Gy) per daily fraction to a total dose of 69.96 Gy (range, 66–77 Gy), 33 (range, 33–35) fraction for PTVnx and PTVnd. Prescribed dose was 59.4 Gy (range, 57.75–63 Gy) to PTV1, 56.1 Gy (range, 52.8–61.25Gy) to PTV2.

Induction and adjuvant chemotherapy regimens included paclitaxel (175 mg/m^2^ d1) + cisplatin (75 mg/m^2^ d1–3), docetaxel (75 mg/m^2^ d1) + cisplatin (75 mg/m^2^ d1–3), docetaxel (60 mg/m^2^ d1) + cisplatin (60 mg/m^2^ d1–3) + 5-fluorouracil (600 mg/m^2^/d d1–5), 5-fluorouracil (1000 mg/m^2^/d d1–5) + cisplatin (75 mg/m^2^ d1–3), every 3 weeks per cycle. Patients received 1 to 4 cycles of IC and 1 to 6 cycles of AC. After 4 weeks when finishing IMRT, AC was administered.

Patients were followed up every 2 to 3 months for the first 3 years, every 4 to 6 months for the next 2 years, and then annually. Overall survival (OS) was defined as the date of histologic diagnosis to the date of death from any cause or last visit. Local-regional control (LRC) rate was computed from the date of histologic diagnosis to the date of the first recurrence in the nasopharyngeal and/or cervical region. Distant failure-free rate (DFFR) was measured from the date of histologic diagnosis to the date of distant failure or last visit. Progression-free survival (PFS) was defined the date of histologic diagnosis to the date of treatment failure or death, whichever occurred first. The Statistical Package for Social Sciences (SPSS version 21.0, Chicago, IL) was used for statistical analysis. The Kaplan–Meier method was used to calculate the OS, LRC, DFFR, and PFS.^[8]^ Multivariate analysis was used to test independent significance of prognostic factors by the Cox hazard model.^[9]^ The *P*-value < .05 was considered to be statistically significant.

## 3 Results

### 3.1 Patient characteristics

Between January 2012 and October 2018, a total of 243 patients were diagnosed as NPC without distant metastasis who were received IMRT treatment in The First Affiliated Hospital of Fujian Medical University. The median follow-up time is 36 months (range: 8–91 months). Table 1 summarizes the demographic characteristics of all patients.

**Table 1:** Characteristics of 243 patients.

		Number	Percent
Sex	Male	184	75.7%
	Female	59	24.3%
Age (yr)	<60	214	88.1%
	≥60	29	11.9%
T	T1	12	4.9%
	T2	71	29.2%
	T3	89	36.6%
	T4	71	29.2%
N	N0	7	2.9%
	N1	31	12.8%
	N2	148	60.9%
	N3	57	23.5%
Stage	III	131	53.9%
	IVA	112	46.1%
Radiotherapy dose (Gy)	<69.96	43	17.7%
	≥69.96	200	82.3%
Chemotherapy regimen	PTX + P^∗^	129	53.1%
	DTX + P^†^	7	2.9%
	PF^‡^	100	41.1%
	TPF^§^	7	2.9%
Chemotherapy cycle^||^	2	11	4.5%
	3	81	33.3%
	4	126	51.9%
	5	16	6.6%
	6	8	3.3%
	8	1	0.4%

AC = adjuvant chemotherapy; DTX + P = docetaxel + cisplatin; PF = 5-fluorouracil + cisplatin; PTX + P = paclitaxel + cisplatin; TPF = docetaxel + cisplatin + 5-fluorouracil.

∗ Paclitaxel + cisplatin.

† Docetaxel + cisplatin.

‡ 5-Fluorouracil + cisplatin.

§ Docetaxel + cisplatin + 5-fluorouracil.

|| IC cycle + AC cycle.

### 3.2 Survival

**Figure 1:**
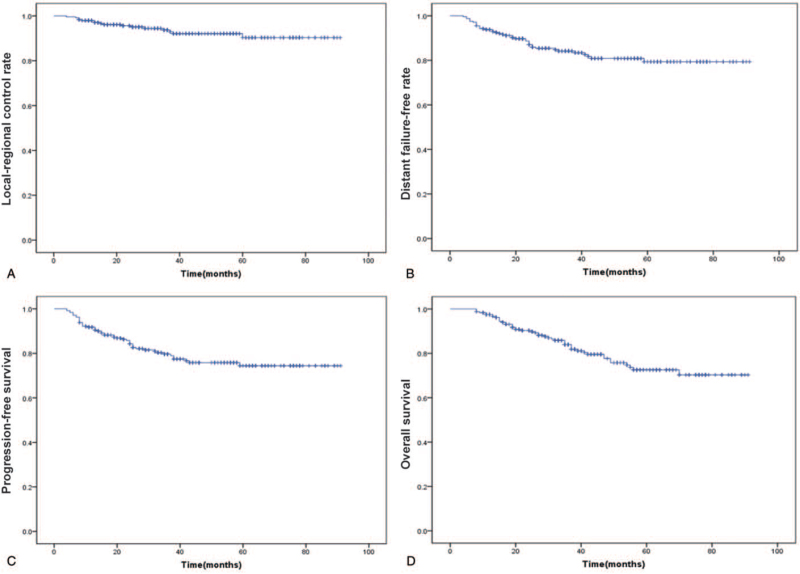
Local-regional control rate (A), distant failure-free rate (B), progression-free (C), and overall (D) survival curves of all patients.

There were totally 47 (19.3%) patients died: the major causative reason is nasopharyngeal carcinoma except 1 patient because of gastric carcinoma and 1 because of car accident. There were 16 out of 243 (6.6%) patients found local-regional recurrence, of which 14 in nasopharyngeal recurrence, while the remaining 2 in cervical region recurrence. Moreover, 39 (16.0%) patients developed distant metastasis. Among these, 34 developed distant metastasis in a single organ (14 cases in lung, 9 in bone, 7 in liver, 2 in brain, 1 in parotid gland, and 1 in retroperitoneum; 5 patients had multiple metastasis (≥2 sites). The overall 3-year LRC, DFFR, PFS, and OS rates were 93.3%, 84.2%, 79.6%, and 84.0%, respectively (Table 2). The 5-year LRC, DFFR, PFS, and OS rates were 90.3%, 79.4%, 74.4%, and 72.6%, respectively (Table 2 and Fig. 1). The patient group who received IC using paclitaxel plus cisplatin shows 5-year rates of LRC, DFFR, PFS, and OS ranging from about 70% to 88% (Table 2). Particularly, patients treated by two-cycle IC combined with IMRT, and subsequently followed by two-cycle AC achieved a higher OS rate compared the former group (74.2% versus 69.7%, Table 2). The 5-year OS rates of patients in stage III and IVA shows significant decreases as compared to that of 3-year (75.1% versus 91.5%, and 56.5% versus 86.5%, respectively, *P*-value < .001; Table 2).

**Table 2:** Treatment outcomes of 243 patients.

	OS (%)		LRC (%)		DFFR (%)		PFS (%)	
No.	3-Year	5-Year	3-Year	5-Year	3-Year	5-Year	3-Year	5-Year
All patients	84.0	72.6	93.3	90.3	84.2	79.4	79.6	74.4
Regimen								
PTX + P	81.9	69.7	90.9	88.0	81.0	75.9	75.7	70.1
Cycle								
4	89.7	74.2	92.5	87.8	89.5	84.1	84.7	79.0
Stage								
III	91.5	86.5	96.2	N/A	89.1	N/A	86.9	N/A
IVA	75.1	56.5	88.7	82.5	77.4	68.2	69.5	61.3

DFFR = distant failure-free rate, LRC = local-regional control, N/A = not applicable, OS = overall survival, PFS = progression-free survival, PTX + P = paclitaxel + cisplatin.

### 3.3 Outcomes of different chemotherapy cycles

The DFFR shows statistically significant for two-cycle versus four-cycle, three-cycle versus four-cycle, and four-cycle versus six-cycle (Table 3). Moreover, comparison between four-cycle and six-cycle shows significant difference in PFS as well (*P*-value = .01; Table 3). In addition, significant difference was also observed in LRC rate of three-cycle versus sis-cycle (*P*-value = .01; Table 3).

**Table 3:** Chi-squared test of different chemotherapy cycles.

	OS		LRC		DFFR		PFS	
	*χ* ^2^	*P*	*χ* ^2^	*P*	*χ* ^2^	*P*	*χ* ^2^	*P*
2 versus 3	0.40	.53	0.55	.46	0.67	.41	0.32	.57
2 versus 4	0.87	.35	0.001	.97	4.07	.04^∗^	2.09	.15
2 versus 5	0.63	.43	0.09	.76	1.13	.29	0.48	.49
2 versus 6	0.26	.61	0.10	.75	0.38	.54	1.09	.30
3 versus 4	1.15	.28	1.45	.23	4.27	.04^∗^	2.68	.10
3 versus 5	0.05	.82	0.86	.35	1.40	.24	0.58	.45
3 versus 6	0.04	.85	6.06	.01^∗^	0.60	.44	2.68	.10
4 versus 5	0.34	.56	0.02	.89	0.05	.82	0.006	.94
4 versus 6	0.54	.46	0.99	.32	4.43	.03^∗^	6.44	.01^∗^
5 versus 6	0.03	.86	0.36	.55	2.28	.13	2.46	.12

DFFR = distant failure-free rate; LRC = local-regional control; OS = overall survival; PFS = progression-free survival.

∗ *P*-value < .05.

### 3.4 Multivariate analysis of prognostic factors

T-category was an adverse prognostic factor that could predicate OS, LRC, DFFR, and PFS, whereas N-category showed significant impact on DFFR and PFS (Table 4). In addition, OS and LRC was also affected by stage and radiation dose, respectively (Table 4).

**Table 4:** Multivariate analysis of prognostic factors.

	OS	LRC	DFFR	PFS
Age	NS	NS	NS	NS
Sex	NS	NS	NS	NS
T	0.036^∗^	0.01^∗^	0.001^†^	<0.001^†^
N	NS	NS	0.017^∗^	0.016^∗^
Stage (III/IVA)	0.03^∗^	NS	NS	NS
Radiation dose	NS	0.006^†^	NS	NS
IC regimen	NS	NS	NS	NS
IC cycle	NS	NS	NS	NS
AC cycle	NS	NS	NS	NS

AC = adjuvant chemotherapy; DFFR = distant failure-free rate; IC = induction chemotherapy; LRC = local-regional control; NS = not significant; OS = overall survival; PFS = progression-free survival.

∗ *P*-value < .05.

† *P*-value < .01.

## 4 Discussion

Our study shows that 2-year LRC, DFFR, PFS, and OS of IC-IMRT-AC that included paclitaxel + cisplatin chemotherapy were 92.7%, 85.9%, 82.9%, and 89.1%, respectively; while IC-IMRT-AC with 5-fluorouracil + cisplatin were 96.7%, 90.5%, 87.3%, and 91.3%, respectively. There were no significant between paclitaxel + cisplatin chemotherapy and 5-fluorouracil + cisplatin chemotherapy (*P*-value = .17). Gemcitabine combined with cisplatin chemotherapy regimen was convenient for usage. In contrast, continuous infusion of 5-fluorouracil for 120 hours is tedious and requires a central venous catheter device, which gives rise to psychological stress and inconvenience for patients. Docetaxel + cisplatin + 5-fluorouracil regimen has certain risks for patients with diabetes or gastric ulcer since patients would receive premedication with oral dexamethasone before using docetaxel. In addition, our report shows that 4 cycles of IC and AC in total were significantly benefit in DFFR compared to 3 cycles (*P*-value = .04). Total 6 cycles versus 4 were also obtained more benefit in DFFR and PFS (*P*-value = .03 and *P*-value = .01). But this is not consistent with 5 cycles, which might account for no significance effect of IC/AC cycle in multivariate analysis (Table 4). Therefore, further investigation on larger NPC population should provide informative clues about the effect of cycle number of IC conjugated with AC on distant metastasis.

Our study provides evidences that the 3-year OS rate of stage III patients was significantly higher than that in IVA patients (91.5% versus 75.1%; *P*-value < .001); and the 5-year OS rates were 86.5% versus 56.5% in the corresponding patients (*P*-value < .001). Wu et al reported a similar result that the 5-year OS rates of stage III and IVA–B disease were 87.0% and 75.5%, respectively (*P*-value = .048).^[10]^ Moreover, stage III showed consistently higher rates in 3-year LRC, DFFR, and PFS (Table 2). Our data also showed the stage III/IVA–B significantly benefits OS rate (Table 4), which is in line with Liu and his colleagues’ study.^[11]^ These data suggests that more advanced stage indicates a worse survival. The combined analyses of NPC-9901 and NPC-9902 trials showed that additional AC with a fluorouracil-containing regimen contributed to the improvement of distant control.^[12]^ However, a meta-analysis showed that the best treatments for locoregional control were IC-RT-AC (P-score 90%).^[2]^ The best treatment for distant control was IC followed by CCRT (P-score 95%).^[2]^ Our trail shows that all the 5-year LRC and DFFR were 90.3% and 79.4% in all patients, respectively, implying that IC-RT-AC is better than locoregional control in therapeutic outcome.

Multivariate prognostic analysis revealed that T-category was a significant factor that can predicate OS, LRC, DFFR, and PFS, while N-category was the factor only for DFFR and PFS (Table 4). Therefore, our IC-RT-AC regimen here demonstrates a significant superiority for the survival of early-stage group as well as distant metastasis of locally advanced group (Table 4). Particularly, PFS was also dramatically impacted by locally advanced stage that was also observed by previous study,^[13]^ highlighting the important role of concurrent chemotherapy with IMTR. Our IC-RT-AC would further benefit those patients with locoregionally advanced stage because NPC in Southern China is the most frequent among China. One study reported that T-category is not a significant prognostic factor for LRC when IMRT is used.^[3]^ This might result from irradiation via IMRT in tumor regions sufficiently improves LRC rate of the NPC patients. Moreover, our data also shows that radiation dosage would positively impact LRC (Table 4). Stage was an interesting prognostic factor that affected the OS rate (Table 4), which is line with the above finding that 3-/5-year OS rates of stage III were significantly higher than corresponding rates of stage IVA (Table 2). Similar observation has also been reported by Au et al.^[14]^ There are several limitations in our study that need to be considered. First, it is a retrospective study and in a single medical center of China. Second, the evaluation data of acute and late toxicities is not available for these patients. Third, DNA detection of Epstein-Barr virus, the major causative virus of NPC,^[15]^ in these patients had not been conducted yet.

## 5 Conclusions

In summary, IC followed by a sequential combination with IMRT and AC for locoregionally advanced NPC is an effective combinatorial treatment. IC-IMRT-AC has superior outcome compared to the locoregional control. Future therapy investigation focusing on distant metastasis would be beneficial for locoregionally advanced NPC treatment.

## Acknowledgments

We thank all those specialists not as co-authors in this study for professional comments and critical thoughts on the manuscript.

## Author contributions

Peirong Wang and Chunlin Ke conceived and designed the study. All authors participated in the acquisition, analysis, and interpretation of data; Peirong Wang and Chunlin Ke drafted the manuscript. All authors read and approved the manuscript.

**Conceptualization:** Peirong Wang, Chuanshu Cai, Chunlin Ke.

**Data curation:** Peirong Wang, Feng Dong, Chuanshu Cai.

**Formal analysis:** Peirong Wang.

**Funding acquisition:** Peirong Wang.

**Investigation:** Peirong Wang, Feng Dong.

**Methodology:** Peirong Wang, Chuanshu Cai, Chunlin Ke.

**Resources:** Peirong Wang, Chunlin Ke.

**Supervision:** Chunlin Ke.

**Writing – original draft:** Peirong Wang, Chunlin Ke.

**Writing – review & editing:** Peirong Wang, Chunlin Ke.
